# Outcomes of poorly differentiated and plasmacytoid variant bladder urothelial carcinoma

**DOI:** 10.1002/bco2.108

**Published:** 2021-08-27

**Authors:** Kathleen Lockhart, Simon King, Alexander Grant, Nicholas McLeod, Albert Tiu

**Affiliations:** ^1^ Department of Urology John Hunter Hospital New Lambton New South Wales Australia; ^2^ Department of Pathology John Hunter Hospital New Lambton New South Wales Australia

**Keywords:** epidemiology, plasmacytoid urothelial carcinoma, urinary bladder neoplasms, urologic surgery

## Abstract

**Objectives:**

The aim of this study is to assess the course and management of poorly differentiated bladder urothelial carcinoma (UC), including plasmacytoid UC (PUC), in our local area. Although bladder cancer is relatively common, PUC is a rare and aggressive subtype with a poor prognosis that is still poorly understood.

**Materials and Methods:**

A retrospective assessment of all poorly differentiated high‐grade UC over the last 15 years (2005–2020) in the Hunter New England area was completed. In total, 37 patients were included, and PUC variant was compared with the remaining poorly differentiated UC.

**Results:**

Of the included cases, eight were PUC, nine squamous variant, two neuroendocrine, and one sarcomatoid. Overall, 23 cases proceeded to cystectomy, 15 had chemotherapy (six neoadjuvant), and 11 had radiation therapy. In the PUC subgroup, three had metastatic disease at diagnosis (37.5%). Of the three PUC patients who underwent cystectomy, all were upstaged. Two PUC cases had adjuvant chemotherapy, and one case had radiation. Within the follow‐up period, the PUC group had a cause‐specific mortality of 50% with a mean survival in these patients of 202 days, compared with 37.9% cause‐specific mortality with survival of 671.55 days (*p* = 0.23) in all other undifferentiated UC cases; 5‐year cause‐specific mortality with Kaplan–Meier analysis was estimated at 26% compared with 59%, respectively (*p* = 0.058).

**Conclusion:**

Poorly differentiated UC is demonstrated to have a poor prognosis with a high mortality rate, particularly when PUC is present. Given the rarity of these variants, further studies are necessary to explore the impact of current treatment options.

## 1 INTRODUCTION

Over 3000 people receive a new bladder cancer diagnosis each year in Australia. In 2019, it was estimated that the risk of an individual being diagnosed with bladder cancer by their 85th birthday will be 1 in 68 (1 in 42 males and 1 in 159 females).^1^, ^2^ Worldwide, bladder cancer is the 10th most common cancer (sixth most common cancer in men) reflected in the 550 000 new cases diagnosed in 2018 and accounting for 2.1% of all cancer deaths.^3^, ^4^, ^5^ Urothelial carcinoma (UC) accounts for 90% of all bladder cancers; of these, approximately 20–30% are invasive at diagnosis.^6^ Invasive UC has a much higher rate of divergent differentiation and therefore often demonstrates histologic variants (in approximately one third of invasive UC).^6^, ^7^ Plasmacytoid UC (PUC) is one of these rare variants, accounting for 1–3% of invasive UC.^6^ It was first described in 1991 and was included in the World Health Organization (WHO) classification of tumors in 2004.^8^, ^9^

**FIGURE 1:**
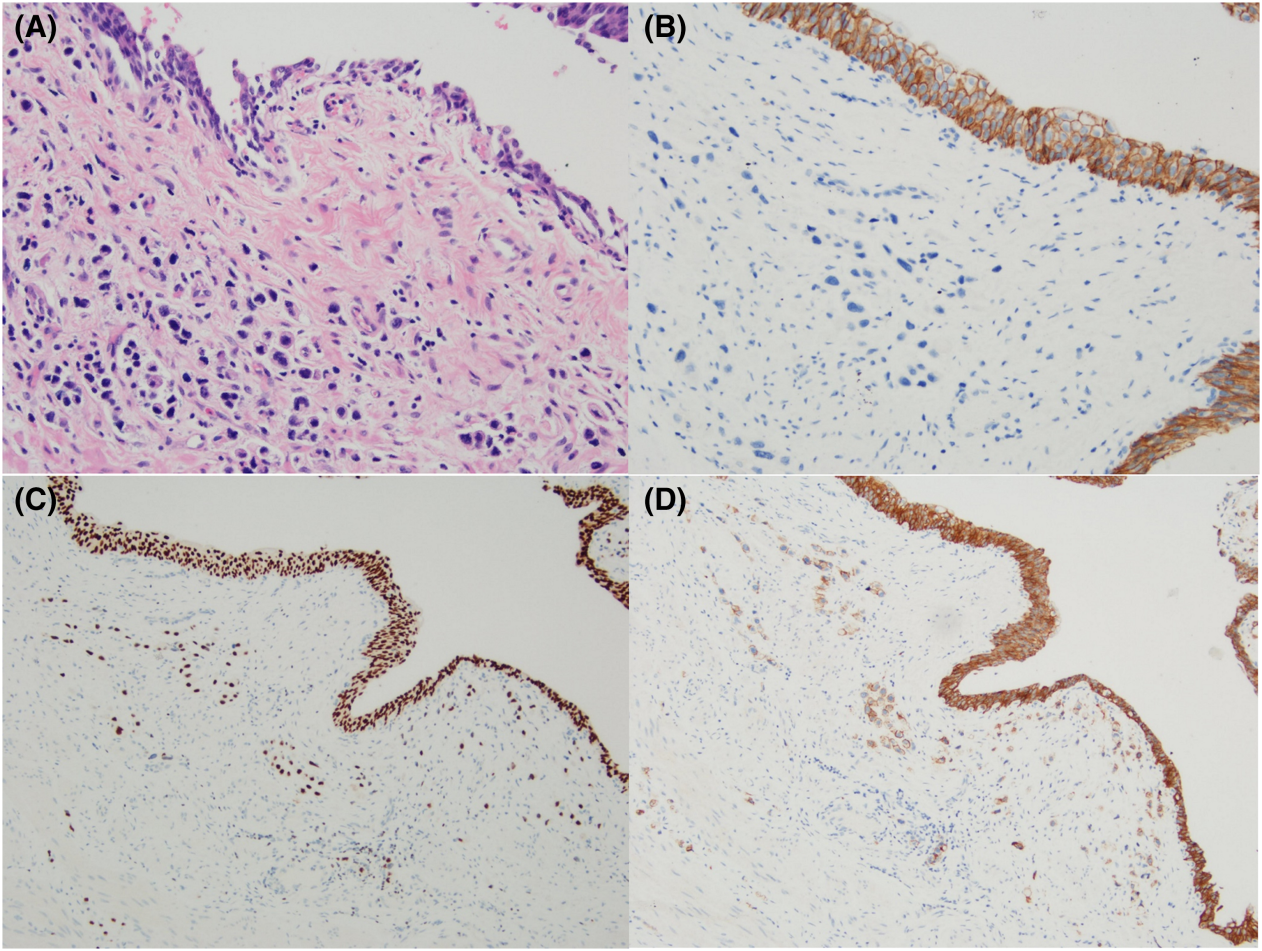
Histopathological features of plasmacytoid urothelial carcinoma: (A) diffuse infiltrate of plasmacytoid urothelial carcinoma undermining benign urothelium (hematoxylin and eosin ×200), (B) E‐cadherin expression is lost within the invasive plasmacytoid urothelial carcinoma cells contrasting with the overlying positive benign urothelium (E‐cadherin ×200), (C) nuclear GATA3, and (D) CD138 membranous expression seen in plasmacytoid urothelial carcinoma and overlying urothelium (×100)

Of all UC variants, it is the most likely to be found in its pure form.^8^, ^9^ PUC is characterized by infiltration of discohesive monotonous small round cells with amphophilic cytoplasm and eccentrically placed round nuclei (Figure 1). This variant of UC resembles plasma cells in appearance and lobular carcinoma of breast and signet ring carcinoma of stomach in both cell appearance and infiltrative architecture, and if no in situ urothelial component is evident, one should also consider metastasis. This differential diagnosis can be difficult to distinguish in this setting because PUC can occasionally express intestinal marker CDX2 and breast markers progesterone receptor and GCDFP‐15.^10^ PUC has a similar immunohistochemical profile to conventional UC with variable expression of GATA3 (80% of PUC cases), P63, CK7 (92%), S100P and CK20 (72%), and uroplakin II (33%), although the diagnosis of plasmacytoid variant is usually made morphologically.^6^, ^8^, ^11^ PUC expresses CD138, which is also a plasma cell marker, although it is not entirely specific and stains other epithelial tumors.^12^ The vast majority of PUCs are distinguished by an additional somatic mutation/promoter hypermethylation affecting the CDH1 gene, which is demonstrated by the loss of E‐cadherin (a protein that maintains intercellular adhesion) and can usually aid in diagnosis of plasmacytoid variant if there is doubt (Figure 1).^6^, ^13^ Occasional E‐cadherin‐positive cases have been reported, which seem to have a better prognosis.^7^, ^14^ Most PUCs also lack expression of the RB gene, which encodes for a tumor‐suppressor protein that regulates cell cycle senescence and apoptosis. RB mutations are more likely to be found in muscle invasive than superficial UCs.^6^, ^7^ HER2 overexpression has also been observed in PUC.^15^

Clinically, PUC has a poor prognosis, characterized by aggressive, invasive cancer and early metastasis with a predilection for peritoneal spread.^9^, ^14^, ^16^, ^17^ Locoregional spread is typically along pelvic fascial planes to involve perivesical, perirectal, and periureteric soft tissues; this can be seen on computed tomography (CT) and magnetic resonance imaging (MRI) as well as during intraoperative inspection.^16^ PUC has a higher frequency of stage ≥pT3 tumors at diagnosis, a higher risk of lymph node metastases, positive margins, and perivesical extension at radical cystectomy than conventional UC.^18^ The recent meta‐analysis published this year by Kim et al. found no difference in cancer‐specific mortality but worse overall survival outcomes.^18^

The mainstay of treatment is radical cystectomy where patient and tumor factors allow, and (neoadjuvant) chemotherapy. Although there is still limited data available regarding PUC response to chemotherapy, it is less responsive than conventional UC in large series. Diamantopoulos et al. reported a ypT0N0 rate after neoadjuvant chemotherapy of 10% in PUC compared with 33% in conventional UC.^14^, ^18^ Nonetheless, some cases have reported responsiveness to typical chemotherapeutic agents.^19^ Usually, platinum‐based chemotherapy is given with regimes such as methotrexate, vinblastine, doxorubicin, and cisplatin (MVAC) or gemcitabine and cisplatin (GC).^14^, ^20^

The aim of this study is to assess the prevalence, treatment, and outcomes of plasmacytoid variant and undifferentiated high‐grade UC of the bladder in our local health district.

## 2 MATERIALS AND METHODS

This was a retrospective study of poorly differentiated high‐grade urothelial cancer with subgroup analysis of plasmacytoid variant in adults (≥18 years) over a 15‐year period (2005–2020) in Hunter New England Local Health District, New South Wales, Australia. It covers a region of 131 785 km^2^ with estimated population of 920 370 people. Local ethics approval was obtained (authorization number: AU202010‐09). All patients with poorly differentiated high‐grade UC of the bladder were included; those with plasmacytoid variant histopathology were compared with all other poorly differentiated high‐grade UC. Histopathological diagnosis was confirmed with immunohistochemistry staining if unclear in all included cases and reviewed by a uropathologist at the time of clinical treatment. Data collection from the medical record included basic demographics, calculated Charlson Comorbidity Index (excluding the new diagnosis of bladder cancer), staging, histopathology results, treatment given including surgical resection, chemotherapy or radiation therapy, metastatic progression, and survival. Outcomes assessed included a comparison of baseline characteristics and prognosis/survival. The overall and cause‐specific survival was compared between PUC and all other included poorly differentiated UC using the Kaplan–Meier method; *p* values less than 0.05 were considered statistically significant.

## 3 RESULTS

A total of 37 patients with high‐grade poorly differentiated UC were included over a 15‐year period from January 2005 to December 2020. Of the included cases, eight were PUC. In the comparison UC group, nine were noted to have squamous variant, two neuroendocrine, and one sarcomatoid, and the remaining were poorly differentiated without identifiable variant pathology. The average age at diagnosis was 71.13 years, and average Charlson Comorbidity Index score was 4.73. Six patients had metastatic disease at diagnosis—three of these patients were plasmacytoid variant (37.5% of this group). All of the patients were discussed in our uro‐oncology multidisciplinary meeting before proceeding to definitive treatment options.

Overall, 23 cases proceeded to cystectomy, 15 had chemotherapy (six neoadjuvant), and 11 had radiation therapy. There were a total of 18 deaths (48.65%) in the study period, with two additional patients lost to follow‐up but metastatic progression demonstrated at last review. Mean overall survival of those who died in the study period was 24.9 months, and mean cause‐specific survival was 18.2 months.

The mean age of diagnosis in the PUC subgroup was 74, with a mean Charlson Comorbidity Index of 5.25 (compared with 4.76 in all other UCs, *p* = 0.54). Of the three patients with metastatic PUC at diagnosis, one had palliative radiotherapy to manage significant hematuria. The remaining two had palliative management. One with pT2 PUC was not deemed suitable (in shared decision making according to patient preference) for surgical intervention or chemoradiotherapy due to comorbidities and had further staging with PET within 3 months demonstrating nodal disease. Another with pT2 disease on diagnosis was found to have concurrent lung cancer on further staging; this took treatment priority, and the patient went on to have palliative chemoradiotherapy (chemotherapeutic agents carboplatin and etoposide). Of the three PUC patients who had a cystectomy, all were upstaged—one had adjuvant palliative chemotherapy for T4b disease and positive margins (six cycles cisplatin and gemcitabine completed 5 months postoperatively), and two went on to have imaging surveillance with Oncology without further chemoradiotherapy to date.

Table 1 compares the characteristics of the PUC group and all other poorly differentiated UC cases. When considered indicated at the time by the reviewing Pathologist, immunohistochemistry profiles of the included PUC cases were performed; these are outlined in Table 2; many were able to be diagnosed predominantly on morphological features.

**TABLE 1:** Comparison of characteristics: Plasmacytoid compared with other poorly differentiated urothelial carcinomas

	Plasmacytoid UC (*n* = 8)	Poorly differentiated UC (*n* = 29)
Age at diagnosis (mean)	74.25	70.28
CCI	5.25	4.76 (*p* = 0.54)
Metastatic disease at diagnosis	3 (37.5%)	3 (10.34%) (*p* = 0.068)
Staged as T2 on initial histopathology (TURBT)	6 (75%)	18 (62.07%) (*p* = 0.504)
Number of cystectomies	3 (37.5%)	20 (69.0%)
Number of deaths (bladder cancer‐specific deaths)	4 (4)	14 (11)

Abbreviations: CCI, Charlson Comorbidity Index; TURBT, transurethral resection of bladder tumor; UC, urothelial carcinoma.

**TABLE 2:** Histopathology immunohistochemistry staining for plasmacytoid urothelial carcinoma tumors

Case number	GATA3	p40	Synaptophysin	AE1/3	CAM5.2	SOX10	TTF1	E‐cadherin loss	PAX8	CK7	CK20	34βE12	β‐Catenin	Pancytokeratin
1														
2	Y	Y	N											
3	Y		N	Y	Y	N	N	Y						
4	Y							Y						
5														
6														Y
7														
8	Y								N	Y	Y	Y	N	

*Note*: Y (yes) indicates staining positive, N (no) indicates staining negative, and no value indicates not tested.

**FIGURE 2:**
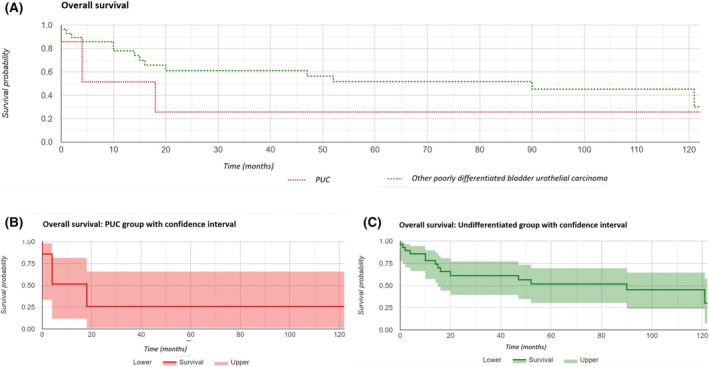
(A) Kaplan–Meier analysis of overall survival, (B) plasmacytoid urothelial carcinoma (PUC) group overall survival with confidence interval, and (C) undifferentiated group overall survival with confidence interval

**FIGURE 3:**
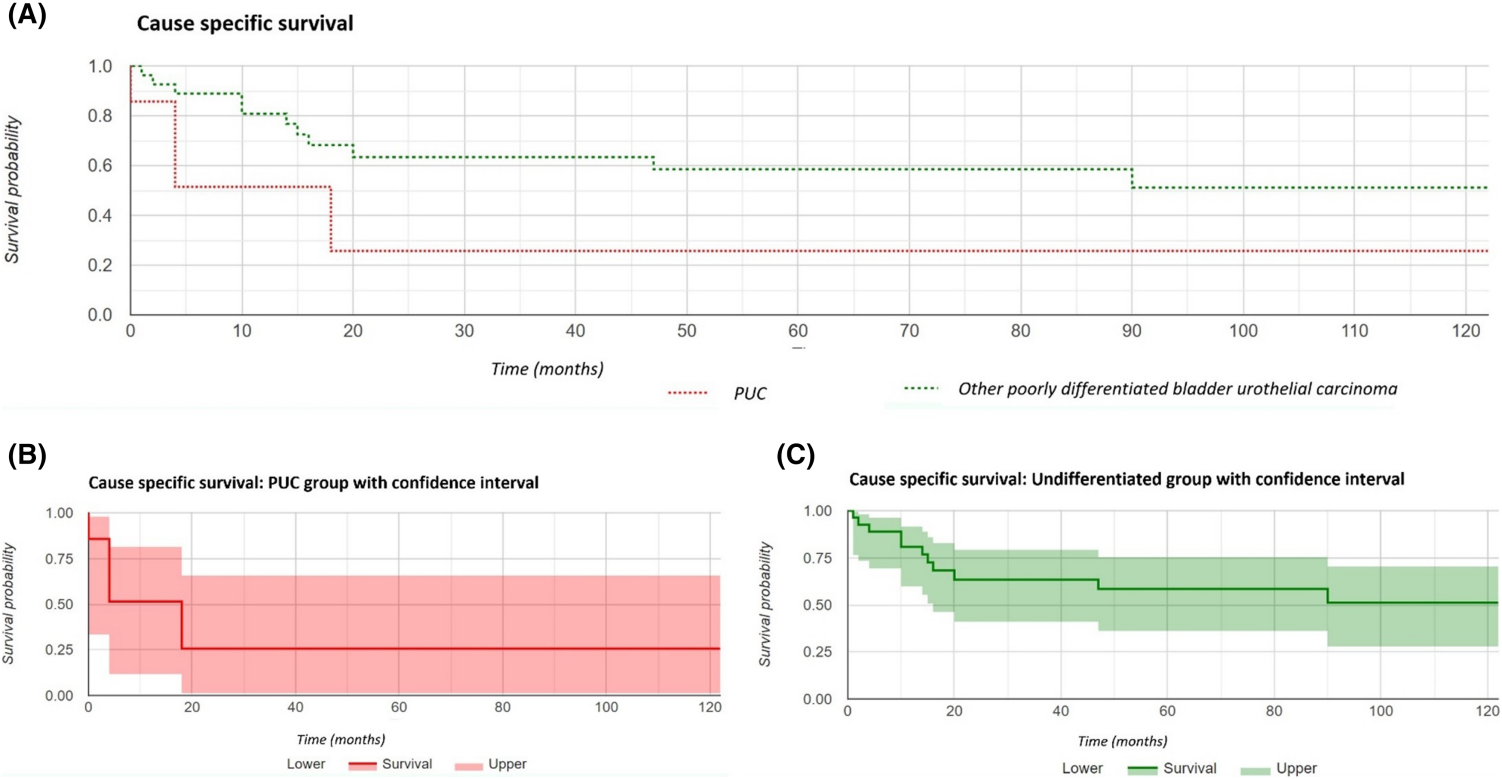
(A) Kaplan–Meier analysis of cause‐specific survival, (B) plasmacytoid urothelial carcinoma (PUC) group cause‐specific survival with confidence interval, and (C) undifferentiated group cause‐specific survival with confidence interval

Within the follow‐up period, the PUC group had a cause‐specific mortality of 50% (four cases; three of these had metastatic disease at diagnosis, and one was upstaged to T4b disease at cystectomy). The mean survival in these patients was 6 months, compared with 37.9% cause‐specific mortality with survival of 22 months (*p* = 0.23) in the poorly differentiated UC group. Notably, there was one further patient in the PUC group who had upstaging to metastatic disease within the study period (apart from the patient who was found to have metastatic concurrent cancer)—suggesting that follow‐up over a longer period would result in even higher mortality rates. A Kaplan–Meier curve analysis was performed; cause‐specific survival demonstrated a higher mortality rate in the PUC group compared with all other poorly differentiated UCs with a 5‐year survival rate of 26% in the PUC group compared with 59% in the undifferentiated group, with borderline statistical significance (*p* = 0.058). Overall survival was not significant but similarly indicated a 5‐year survival of 26% in the PUC group compared with 52% in the undifferentiated group (*p* = 0.103). Overall survival and cause‐specific survival Kaplan–Meier curves are included as Figures 2 and 3.

## 4 DISCUSSION

PUC has a poor prognosis, with a number of series describing 5‐year survival rates from 8% to 40% for stage T3 or higher tumors.^19^ Radical cystectomy is thought to have survival benefit, although there is high risk of selection bias with patients at higher staging and comorbidity status less likely to be suitable for surgical management.^14^ Poorly differentiated high‐grade UCs with aggressive variants like PUC have higher rates of early metastasis, resulting in a lower proportion of patients who are suitable for surgical management. These findings are consistent with our results, with a higher proportion of PUC cases metastatic at diagnosis and >pT2 at endoscopic diagnosis, and 37.5% proceeding to cystectomy compared with 69.0% in the undifferentiated group. The response of PUC to chemotherapy and radiation therapy is still not well understood, but it is clear that this aggressive variant invades and metastasizes quickly. As a result, it is vital to ensure earliest possible detection and early treatment. In this series, neoadjuvant chemotherapy was not offered for PUC in this cohort as the current literature has favored surgical resection as the clinical priority if indicated. Adjuvant treatment options were determined by patient and pathology factors.

The major limitations of this study are its retrospective nature, risk of selection bias, and limited size sample. PUC has only been recognized as a UC variant since 2004, so the terminology is relatively new, and in the first few years after this change to the WHO classification of tumors, it may have been underreporte. Our first plasmacytoid case in this study was identified in 2015; this may reflect selection bias for this reason.

Given this variant has propensity for insidious stromal spread, it can also be easily missed on frozen sections and endoscopic bladder resection specimens (particularly if compounded by lack of a typical tumor mass evident endoscopically).

Due to the rarity of these variants and resulting low population numbers, further multicenter prospective studies with standardized inclusion criteria would be beneficial in determining the most effective approach to treatment particularly when it comes to chemotherapy options and adjunct radiotherapy. Early surgical management with radical cystectomy is currently the accepted gold standard for PUC, with associated systemic therapy, typically MVAC chemotherapy.^19^ Nonetheless, it is clear in the literature, and supported by our findings, that PUC has a very poor prognosis despite available treatment options.

In conclusion, poorly differentiated UC in our locality is demonstrated to have a poor prognosis with a high mortality rate, particularly when PUC variant is present even compared with other high‐grade poorly differentiated bladder UCs. Due to the rarity of poorly differentiated UC, further studies will require multi‐institutional collaboration to power a sample large enough to inform its optimal treatment options. Until then, it is crucial to make treatment decisions on a case‐by‐case basis within the context of a multidisciplinary team.

## CONFLICT OF INTERESTS

All authors declare that they have no conflicts of interest or additional sources of support or funding to declare.

## AUTHOR CONTRIBUTIONS

K. L. conceived the study and researched the literature, obtained ethics approval, performed data collection and analysis, and drafted the manuscript. S. K. contributed to the literature review and discussion and some data review. A. G., N. M., and A. T. contributed to the data collection, supervised the study, and reviewed and approved the final manuscript. Ethical approval was obtained from the Hunter New England Health research ethics and governance office.
